# High-power amplified spontaneous emission pulses with tunable coherence for efficient non-linear processes

**DOI:** 10.1038/s41598-021-83443-2

**Published:** 2021-03-01

**Authors:** Nicolas Valero, Denis Marion, Jerome Lhermite, Jean-Christophe Delagnes, William Renard, Romain Royon, Eric Cormier

**Affiliations:** 1 Centre Lasers Intenses et Applications (CELIA), Université Bordeauxp-CNRS-CEA, UMR5107, 351 Cours de la libération, 33405 Talence, France; 2 Institut Universitaire de France (IUF), 1 rue Descartes, 75231 Paris Cedex 05, France; 3 Laboratoire Photonique, Numerique et Nanoscience (LP2N), Institut d’Optique Graduate School-CNRS-Universite Bordeaux, rue F. Mitterrand, 33400 Talence, France; 4 IRISIOME, rue F. Mitterrand, 33400 Talence, France

**Keywords:** Optical physics, Optical techniques

## Abstract

We report on a detailed study of an amplified spontaneous emission source operated in a pulsed regime with particular attention paid to the influence of high-intensity chaotic temporal events on the generation of nonlinear processes. To this aim, we have developed a monolithic high-power fiber system delivering partially coherent pulses of adjustable coherence. We also have demonstrated a non-linear method to characterize the stochastic properties of the source mitigating the bandwidth limitation of linear techniques. Measured parameters of the source for various configurations are presented. An enhanced classical model has been established to reproduce the statistical properties of the source and predict the behaviour when exciting non-linear processes. Finally, a non-linear process (second harmonic generation) is investigated comparing the efficiency when the process is pumped by a pulsed beam with maximal and low coherence.

## Introduction

In march 1917, A.Einstein formulated the interaction theory of light with atoms. Based on phenomenological^1^ and quantum considerations^2^, this theory allows to describe elementary laser physics and in particular the elementary processes underlying Amplified Spontaneous Emission (ASE)^3^. According to Einstein’s theory, physical properties of light sources based on ASE are characterized by electric field (or intensity) temporal fluctuations around a mean value. These sources can be considered as chaotic light sources as for instance “thermal light” sources or light emitted by a free running laser cavity with a very large number of longitudinal modes^4,5^. A typical characteristic of these stochastic sources is the low temporal coherence with a photon distribution following the Bose–Einstein statistic^6–8^. Since decades, researchers have investigated the chaotic aspect of this form of light where fluctuations occur randomly creating high-intensity short temporal events^3^. ASE sources in the continuous regime^5,9–14^ are very interesting examples of such stochastic light. They have been successfully used in many applications^15–20^ including low-coherence interferometry, sensing, imaging, speckle free imaging, low-coherence tomography, entanglement of photons pairs^21^ and enhanced non-linear optical generation^12,22,23^.

In 2014, a Japanese team^22^ studied the enhancement of second harmonic generation (SHG) in a nonlinear crystal using a few mW CW ASE low-coherence pump beam centered at 1551 nm wavelength with different spectral bandwidths. They have demonstrated that the use of a CW ASE pump improves the SHG conversion efficiency by a factor of two in comparison to pumping with a fully coherent beam, as predicted by the quantum theory of light^3^. However, the use of a CW ASE source may reveal its limitation in cases involving higher nonlinear effects or even cascaded nonlinear effects requiring high-peak power accessible only with energetic pulses^15^.

In this paper, we report, for the first time to the best of our knowledge, on a study of an ASE source operated in a picosecond pulsed regime and focusing on the influence of high intensity random events on the generation of nonlinear processes. To this aim, we have developed a monolithic high-power fiber system delivering partially coherent pulses of ASE. The system includes a CW ASE seeder, spectral filtering stages, amplifiers and an electro-optical pulse shaper module. This approach provides a versatile tool where many parameters can be adjusted by the user: central frequency, average power, pulse repetition rate, pulse energy, pulse duration and most importantly the coherence time. We also have developed a non-linear method to characterize the stochastic properties of the source. The source is then fully characterized for various configurations. Finally, a non-linear process (SHG) is investigated comparing the efficiency when pumped by a pulsed beam with a maximal and a low coherence.

## Pulsed ASE source statistical properties

### Continuous ASE source modeling

The stochastic properties of an ASE light source can be described by a statistical model. It has been established from a quantum-mechanical approach, that the measured photon-count probability distribution corresponds to a *M*-fold degenerate Bose–Einstein distribution^6^. Here, the degeneracy factor *M* represents the number of coherence temporal cells influencing the signal captured by the receiver. As expected, *M* will depend on the receiver response time compared to the actual coherence time of the source or equivalently on the electrical bandwidth $\beta _{el}$ of the detection system and the optical bandwidth $\beta _{op}$ of the ASE source. Thus, the probability to detect *n* photons during an integration time *T* reads:

1 $$\begin{aligned} P(n,{\overline{n}},M)=\frac{\Gamma (n+M)}{\Gamma (n+1)\Gamma (M)}\left( 1+\frac{M}{{\overline{n}}}\right) ^{-n}\left( 1+\frac{{\overline{n}}}{M}\right) ^{-M} \approx \frac{M^M}{{\overline{n}}\ \Gamma (M)}\left( \frac{n}{{\overline{n}}}\right) ^{M-1}\exp \left( -\frac{M\cdot n}{{\overline{n}}}\right) \end{aligned}$$

for large values of ${\overline{n}}$, where ${\overline{n}}$ is the mean recorded photon number and $\Gamma ($x) is the gamma function. Note that *n* and ${\overline{n}}$ are proportional to the instantaneous laser intensity *I*(*t*) and mean intensity $I_{mean}$ respectively. Thus, $P(n,{\overline{n}},M)$ will be noted *P*(*I*) from now on. The degeneracy factor *M* tends to 1 for $\beta _{op}\ll \beta _{el}$ on the one hand and converges toward $\beta _{op}/\beta _{el}$ for $\beta _{op}>10\ \beta _{el}$ on the other hand. However, the typical values found in our experiment lie in the intermediate range where $\beta _{op}$ is comparable to $\beta _{el}$. In such a case, if we assume a chaotic light source with a polarized Gaussian power spectral density, M can be defined as^6,8^ :

2 $$\begin{aligned} M = \frac{\pi \left( \frac{\beta _{op}}{\beta _{el}}\right) ^2}{\exp [-\pi (\beta _{op}/\beta _{el})^2]-1+ \pi \beta _{op}/\beta _{el} {{\,\mathrm{erf}\,}}(\sqrt{\pi }\beta _{op}/ \beta _{el})} \end{aligned}$$

With erf ,the error function, also called the Gauss error function. For unpolarized ASE the degeneracy factor is doubled with respect to the polarized case.

**Figure 1:**
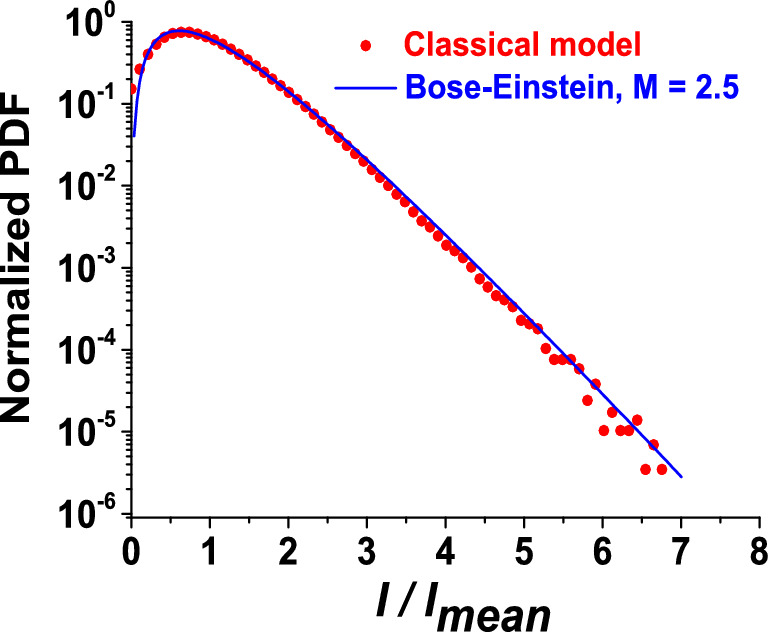
Normalized simulated PDFs of a polarized ASE source. Blue line: PDF simulated with Bose–Einstein theory for M = 2.5. Red dots: PDF simulated with the enhanced classical model.

However, while the quantum mechanical approach of Bose–Einstein distributions allows a good description of the photons statistics, the model excludes access to the time dependence of the amplitude and phase and consequently the intensity of the field. To circumvent this limitation, and being able to predict the propagation in non-linear media, we have implemented a classical model able to reproduce the temporal fluctuations of the ASE source. The model considers the source as a completely stochastic radiation resulting from the incoherent superposition of a large number of discrete spectral modes^24^ covering the ASE spectrum. In contrast to a laser radiation generated in an optical cavity, an ASE source provides a continuous spectrum. In order to mimic the latter property our model discretizes the field frequency distribution with a very high longitudinal mode density. Additionally, each discrete ’mode’ is affected with a randomly chosen phase. Finally, the model is refined by including the limited detection bandwidth of the temporal receiver (see “Methods” for a description of the enhanced classical model).

Figure 1 shows a simulation of the normalized probability density function (PDF) computed using the Bose–Einstein model (1) and the enhanced classical model for a typical case of our experiment. The PDF is plotted as a function of the measured ratio $I(t)/I_{mean}$ (where *I*(*t*) is distorted by the receiver response time). The very good match indicates that the classical model reproduces accurately the stochastic properties of the ASE source. Here, the spectral bandwidth is 60 pm at $\lambda _0$ 1030 nm ($\beta _{op}$ = 16.9 GHz) and the electrical bandwidth $\beta _{el}=7.9$ GHz leading to a ratio $\beta _{op}/\beta _{el}$ of 1.95 and a degeneracy factor *M* 2.5.

### Coherence of a pulsed ASE source

Coherence properties of light fields are essential parameters to describe stochastic light sources from theoretical and experimental points of view. These properties are well defined by two temporal continuous correlation functions^3,4^. First, assuming an ergodic stochastic process, the degree of first order coherence (or field temporal correlation function) is defined by:

3 $$\begin{aligned} g^{(1)}(\tau ) =\frac{\langle E(t)E^*(t+\tau )\rangle }{\langle E(t)E^*(t)\rangle } \end{aligned}$$

where $\tau$ is a delay, *E*(*t*) is the complex electrical light field. The brackets denote time averaging. In addition, according to the model corresponding to a Doppler broadened chaotic source (polarized)^3,6^ with a Gaussian spectral distribution, $g^{(1)}(\tau )$ and the coherence time $\tau _{c}$ can be defined as:

4 $$\begin{aligned} g^{(1)}(\tau )= & {} e^{-iw_{0}\tau }e^{-\frac{\pi }{2}(\frac{\tau }{\tau _{c}})^2} \end{aligned}$$

5 $$\begin{aligned} \tau _{c}= & {} \int _{-\infty }^{\infty } |g^{(1)}(\tau )|^2 \, \mathrm {d}\tau \end{aligned}$$

where $w_{0}$ is the central frequency of the source. As the spectrum of the source and the first order correlation function are related through the Wiener–Khintchine theorem, one can deduce the source bandwidth from the coherence time. In the case of a Doppler broadened source the full width at half maximum bandwidth is given by $\Delta \omega =2\sqrt{\pi }/\tau _{c}\sqrt{2log(2)}$ or equivalently for narrow bandwidth by $\Delta \lambda =\lambda ^2/(2\pi c) \Delta \omega$.

Then, defining the instantaneous intensity as $I(t)=E(t)E(t)^*$, the degree of second order coherence (or intensity temporal correlation function) is given by:

6 $$\begin{aligned} g^{(2)}(\tau ) =\frac{\langle I(t)I(t+\tau )\rangle }{\langle I(t)\rangle ^2} \end{aligned}$$

The function $g^{(2)}(\tau )$ is a major quantity which discriminates chaotic from coherent sources. It has been demonstrated that for a continuous chaotic light source with a large number of emitters and consequently for a large number of photons, the following simple equation holds between the first and second order coherence functions^3,4^:

7 $$\begin{aligned} g^{(2)}(\tau ) = |g^{(1)}(\tau )|^2+1 \end{aligned}$$

Additionally, one can show that $g^{(2)}(\tau )$ obeys the inequalities $1\le g^{(2)}(0)$ and $g^{(2)}(\tau )\le g^{(2)}(0)$^25^. Finally, $g^{(2)}(\tau )$ is limited by $g^{(2)}(0)=2$ and $g^{(2)}(\tau )\rightarrow 1$ when $\tau \gg \tau _c$^3^.

**Figure 2:**
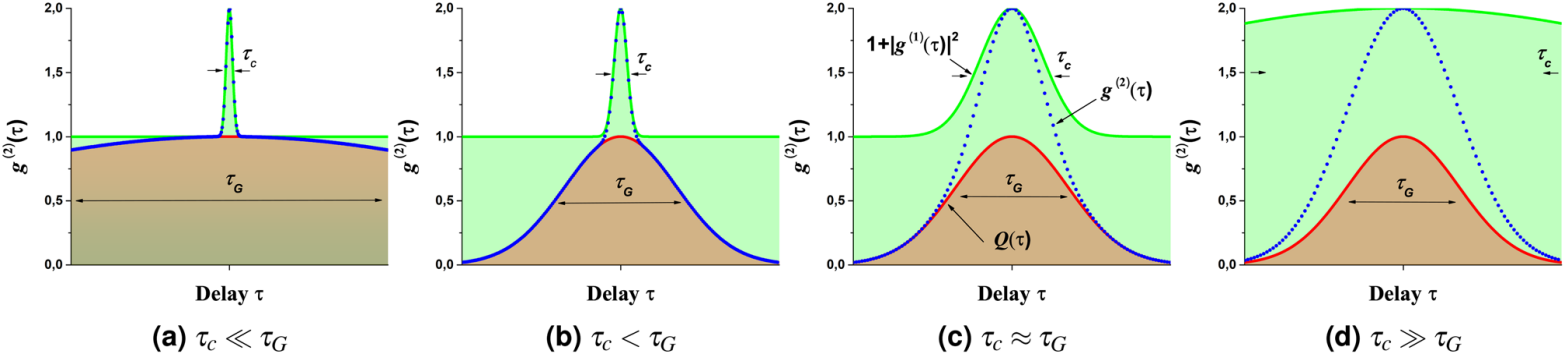
Degree of second order coherence $g^{(2)}(\tau )$ (blue dots) of a pulsed ASE source and its decomposition in the product of the gate intensity correlation function $Q(\tau )$ (red curve) and $1+|g^{(1)}(\tau )|^2$ (green curve) involving the squared degree of first order coherence for various temporal gate durations. Simulated datas with classical model.

The above description applies to a continuous stochastic source. However, the present work addresses the properties of an ASE source in which pulses have been carved (shaped by a temporal gating technique) leading to a chaotic temporal waveform evolving now under a pulse envelop. The coherence properties of such a source are expected to be affected by the gate parameters such as the pulse duration $\tau _G$ and/or the pulse shape. It can be shown that in the case of a pulsed stochastic source, the second order coherence function reads (see complementary materials):

8 $$\begin{aligned} g^{(2)}(\tau ) = Q(\tau ) \left( |g^{(1)}(\tau )|^2+1\right) \end{aligned}$$

where $Q(\tau )$ is the normalized intensity correlation function of the temporal gate with 0 $\le Q(\tau ) \le$ 1. We report on Fig. 2 the typical coherence functions associated with a pulsed ASE source for various coherence time $\tau _c$ and gate duration $\tau _G$ and its decomposition in the product of the gate intensity correlation function $Q(\tau )$ and $1+|g^{(1)}(\tau )|^2$. Figure 2a refers to a very long pulse and converges toward the typical behaviour of a continuous stochastic source where $g^{(2)}(\tau )$ is identically equal to 1 for non-zero delays. The peak observed near the zero delay is referred to as a coherence peak in the literature^26,27^. Its width is related to the coherence time $\tau _c$ of the source. It is a direct manifestation of the temporal coherence degradation and the existence of intensity fluctuations of the source resulting from stochastic photon bunching^4^. Figure 2b reveals the pulsed nature of the ASE source and corresponds to the experimental cases reported here where the gate is longer than the coherence time. The second order coherence shows two main features. On the one hand, the correlation vanishes for delays greater than the pulse duration following $Q(\tau )$ which is the intensity correlation function of the gate whose width depends on the gate duration $\tau _G$. On the other hand, the coherence peak is still present and inform us on the existence of field fluctuations within the pulse envelop. Note that in this very case the decomposition in the product of $Q(\tau )$ containing information on the gate and $1+|g^{(1)}(\tau )|^2$ characterizing the ASE source through the first order coherence is straightforward. When $\tau _c$ is of the order of $\tau _G$ (Fig. 2c), the number of temporal structures leading to intensity fluctuations within the pulse envelop is very limited and the coherence peak becomes indistinguishable from the gate correlation function. Finally, as $\tau _c$ gets greater than $\tau _G$, the ASE bandwidth is too narrow to allow any temporal structure to occur within the pulse duration. However, in this case the energy of the pulses carved in the continuous ASE source will follow a stochastic distribution.

By applying the decomposition explained above, $\tau _{c}$ can be directly extracted from $g^{(2)}(\tau )$ either by a fit on Eq. (4) or simply through the integration in Eq. (5).

### Enhancement of nonlinearities

Perturbation theory to the first order predicts that a process induced by a field of intensity *I* interacting with a non-linear medium with a *n*-th order susceptibility $\chi ^{(n)}$ varies like $I^{n}$. Compared with a monochromatic source of same average intensity $I_{mean}$, a stochastic source can provoke more efficient nonlinear coupling due to the random occurrence of high-intensity temporal structures. Indeed, considering a Bose–Einstein distribution *P*(*I*) for light sources based on ASE (Eq. 1), the enhancement of nonlinear coupling for processes of order *n* scales (if $M \approx 1$ as:

9 $$\begin{aligned} \int _{0}^{\infty } \left( \frac{I}{I_{meam}}\right) ^n P(I) \mathrm {d}I = \int _{0}^{\infty } \left( \frac{I}{I_{meam}}\right) ^n\frac{1}{I_{meam}}\exp \left( \frac{-I}{I_{meam}}\right) \mathrm {d}I = \Gamma (n+1) =n!, \qquad \forall \text {n} > 0 \qquad \end{aligned}$$

where $\Gamma$ is the gamma function^8^. Equation 9 clearly shows that a sizable increase of order *n*! can be expected using ASE sources instead of coherent sources of equivalent mean intensity. Considering for instance the process of second-harmonic generation, a factor 2 can typically be expected. The enhancement of nonlinear coupling with incoherent light is already well-known^28,29^ but suffers limitations that have been investigated in the literature for many nonlinear processes^28–31^. The propagation of high-intensity peaks in fibers and nonlinear crystals can deteriorate this figure, as Eq. (9) also stands for nonlinear processes in these media. Self-phase modulation coupled to dispersion can significantly affect the high intensity temporal structures, hence reducing the enhancement factor. Let us also emphasize that Eq. (9) excludes saturation and phase-matching conditions and therefore should be considered as a maximal value.

## Experimental set-up

**Figure 3:**
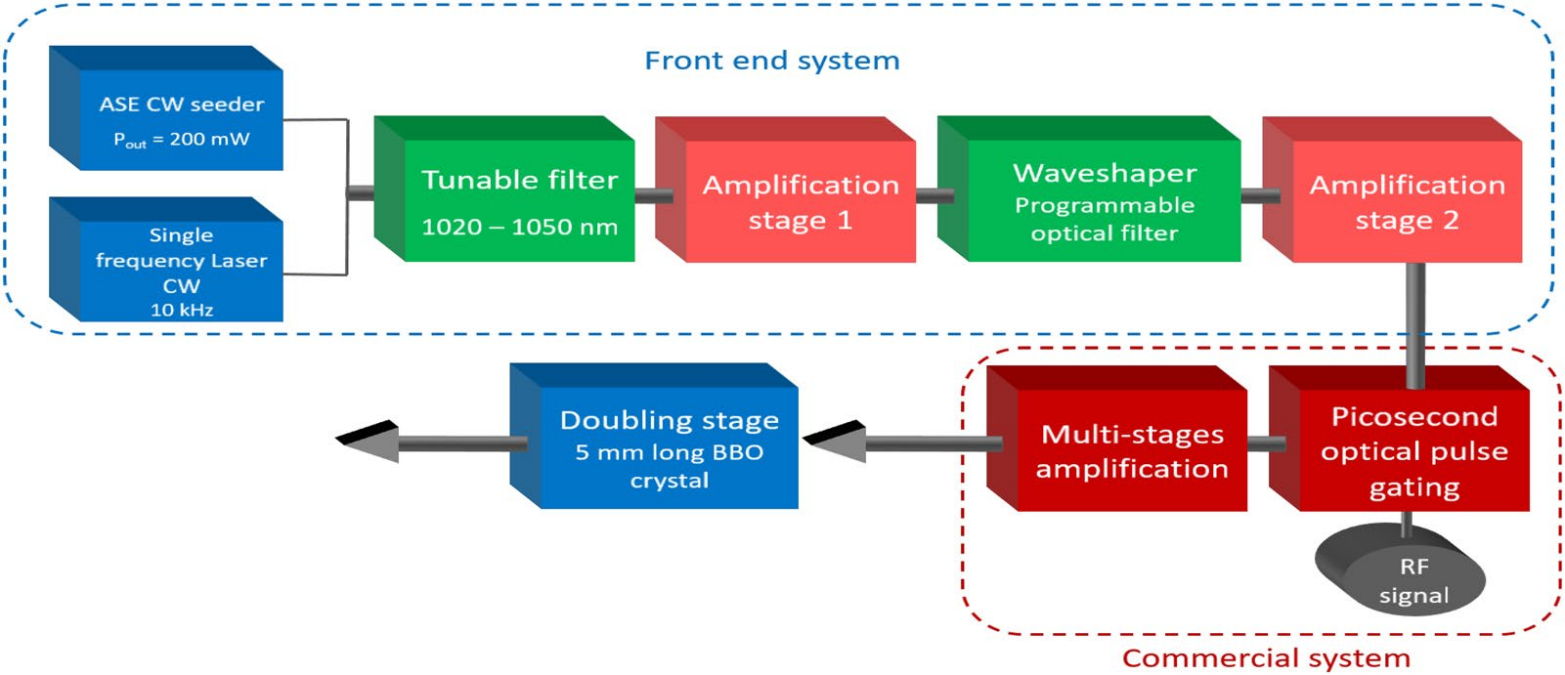
Experimental set-up of the pulsed ASE system delivering up to 20 W of average power.

**Figure 4:**
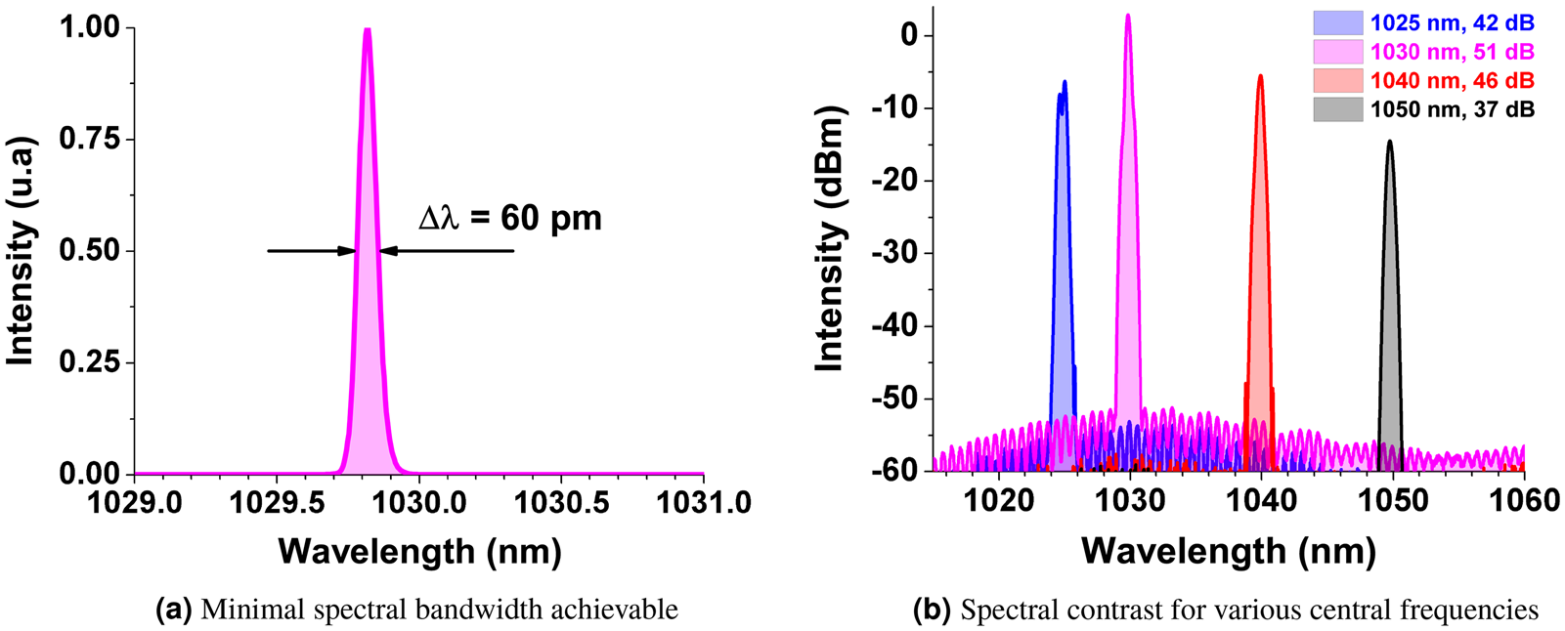
Narrow band configuration of the CW ASE seeder.

The experimental setup of our all polarization maintaining fiber laser system is depicted on Fig. 3. The system consists of four cascaded optical processing modules: a switchable front end comprising either a continuous wave ASE seeder or a single frequency laser, spectral filtering modules combined with a set of low power amplifiers, a picosecond optical pulse generator with high power amplifier stages and a free space frequency doubling stage. The single frequency laser is a Koheras (NKT-Photonics) with 10 kHz optical bandwidth. The continuous wave ASE seeder includes a 10/125 $\upmu$m Ytterbium-doped fiber end pumped through a $2+1 \rightarrow 1$ pump combiner by a 976 nm multimode laser diode delivering up to 10 W of average power. To warrant spontaneous emission operation and avoid any parasitic lasing effects, one end of the active fiber is angle cleaved and the other one is spliced to a fibered isolator. A continuous radiation is delivered with up to 1 W average power at 1030 nm with a spectral bandwidth of 40 nm (11 THz) if injected by the ASE seeder or a bandwidth of 10 kHz if injected by the single frequency laser.When operated with the ASE seeder, a fibered tunable optical filter selects a spectral band with a minimal bandwidth of 1 nm at any central wavelength between 1020 and 1060 nm with an extinction ratio of 30 dB. The losses induced by the filtering are compensated by a core-pumped amplifier based on a 6 $\upmu$m core-diameter polarization-maintaining Ytterbium-doped fiber delivering up to 150 mW of average power. An additional high resolution programmable filter (Waveshaper 1000 A from Finisar) is then inserted that allows to select a narrower spectral bandwidth adjustable from 1 nm down to 60 pm (see Fig.  4a) over the entire emission range with an extinction ratio greater than 35 dB (50 dB close to 1030 nm) as shown in Fig. 4b. The induced losses are compensated by a core-pumped amplifier delivering up to 200 mW.

**Figure 5:**
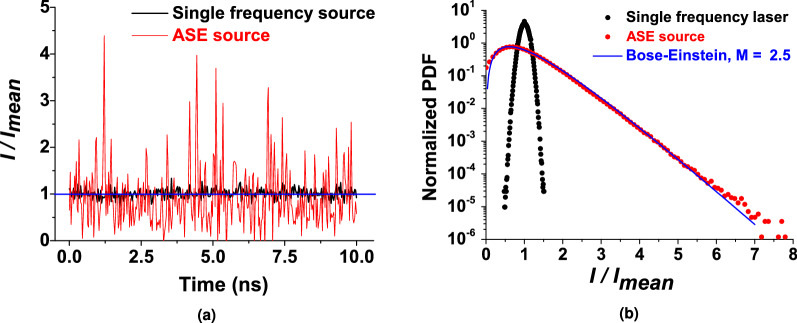
(**a**) Sampled ASE temporal intensity trace from the ASE seed source (red curve) and from the single frequency laser (black curve) normalised to the mean intensity (blue line). (**b**) PDF of the experimental ASE signal (black dots) and the single frequency laser (red dots) together with the Bose–Einstein distribution model with $M = 2.5$ (blue line).

**Figure 6:**
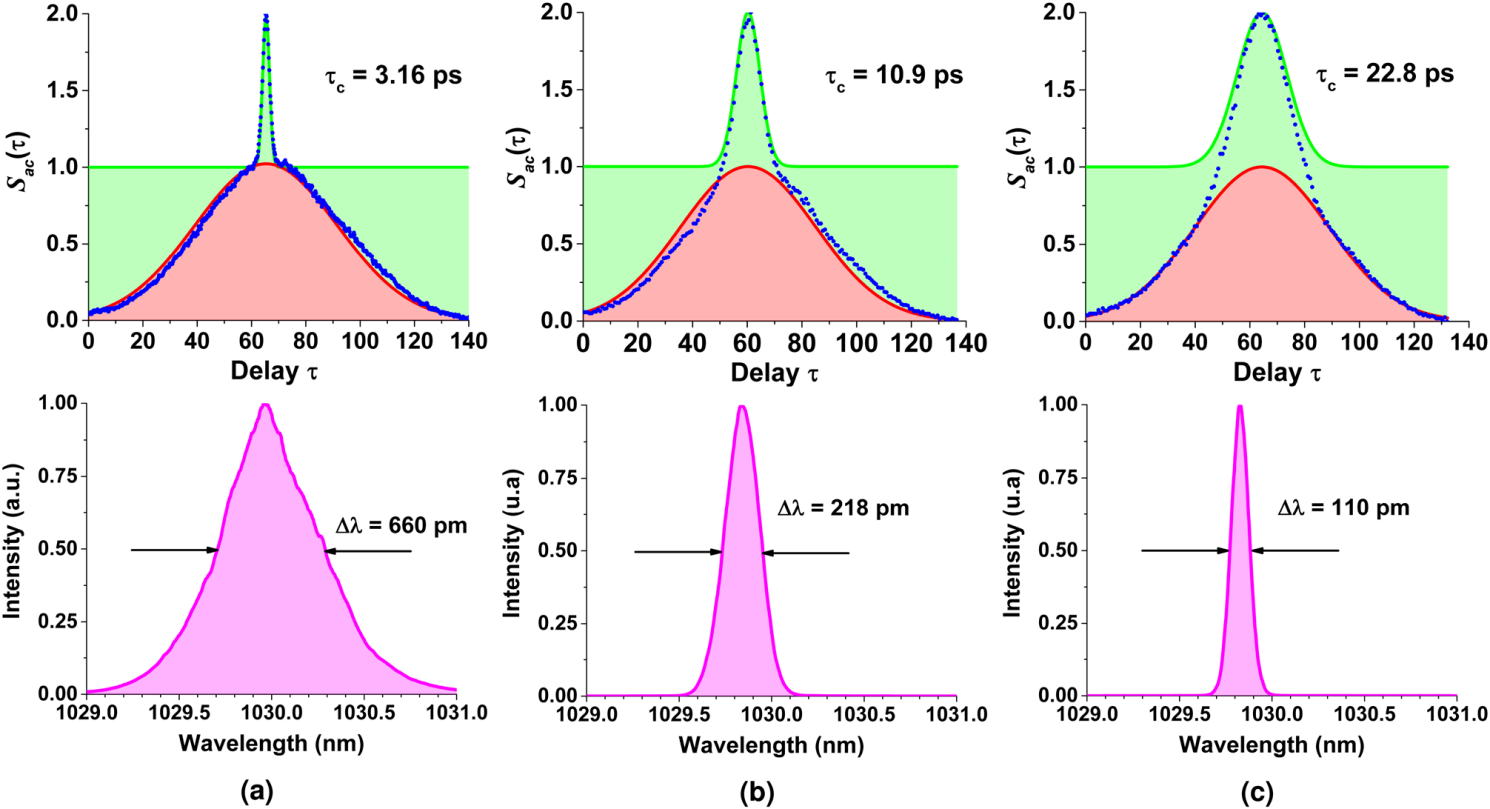
Top panels: experimental normalized autocorrelation traces of the pulsed ASE source for 3 different coherence time. The green and red curves correspond to the extracted first order correlation function $\left( 1+|g^{(1)}(\tau )|^2\right)$ and the gate autocorrelation $Q(\tau )$ respectively. The pulse duration is in this case 42 ps. The coherence time $\tau _{c}$ varies from 3.2 to 22.8 ps. Bottom panels: measured corresponding spectra.

The filtered continuous ASE seed signal is further injected in a commercial device (Manny IR Series from IRISIOME Solutions) able to carve and amplify a train of pulses with a duration as short as 50 ps. The pulse shaping is achieved by means of an electro-optic amplitude modulator driven by a high bandwidth radio-frequency pulsed signal. The pulse repetition rate can be continuously adjusted from 5 MHz to 1 GHz^32–34^. The generated ASE pulsed signal is then amplified through two amplification stages to reach up to 20 W of average power. The last stage consists in a frequency doubling module built around a 5 mm long type I BBO crystal. The fundamental signal centered at 1030 nm is focused by a 400 mm focal length lens leading to a 100 $\upmu \text {m}$ spot diameter in the crystal. CW ASE temporal traces (Fig. 5) were recorded by a 40-GHz InGaAs PIN photodetector connected to a 8.4-GHz real-time oscilloscope. The overall RF bandwidth of the detection system reaches up to 7.9 GHz. In the experiments, the ASE power on the receiver was set to $\approx$ 2 mW, to warranty operation in the linear regime. The Optical spectra (Figs. 4a and 6) were recorded using an Optical Spectrum Analyser with an effective resolution of 46 pm. Infrared and green mean powers (before and after the doubling stage) were recorded using thermopile powermeters.

## Results and discussion

### Linear detection

The first experiment consisted in characterizing the CW ASE signal emitted by the spectrally filtered seed module. We have recorded, on the oscilloscope, temporal traces of the signal received by a photodiode (linear detection). A sample over 10 ns (randomly selected in a 500 $\upmu$s trace) is displayed in Fig. 5a where the instantaneous intensity expresses its chaotic behaviour with clear high-peak intensity temporal structures reaching several times the average intensity but also decreasing to zero intensity. To better appreciate the stochastic field evolution, we also plot a sample trace of the single frequency laser where only small fluctuations around the mean intensity are visible. In order to characterize the ASE source, we have computed the PDF associated to the recorded temporal traces as displayed on Fig. 5b for both the ASE source and the single frequency laser. It should be noted here that the measurements performed with the “linear detection” setup is obviously biased by the finite response of the detection system^10^. In fact, typical optical bandwidth ($\beta _{op}$) are of the order of tens to hundreds of GHz exceeding easily the electrical bandwidth $\beta _{el}$ of the measurement devices (100 GHz for state-of-the-art real-time oscilloscopes) with the consequence that the fastest fluctuations are averaged by the detection system. Thus, the comparison of our measurements with a theoretical Bose–Einstein distribution should account for the experimental limitations as detailed in “Continuous ASE source modeling” through the degeneracy factor *M*. In the present case, the spectral filtering is set to 60 pm ($\beta _{op}$= 16.9 GHz) and the entire detection chain bandwidth is limited to $\beta _{el}$= 7.9 GHz, leading to a ratio $\beta _{op}/\beta _{el}$ of 1.95 and a degeneracy factor $M\approx 2.5$ according to Eq. (2). The theoretical Bose–Einstein distribution (normalized Eq. (1)) corresponding to the experimental conditions is reported on Fig. 5b with an excellent match.

Now, we aim here to shape 50 ps pulses in the continuous ASE signal. In order to make sure wide fluctuations still occur within the pulse envelop (some stochastic behaviour is still preserved), the spectral bandwidth should be increased significantly. Considering one of the case presented below where the ASE bandwidth is set to approximately 700 pm, the electrical bandwidth required to properly analyse the ASE properties (i.e. achieving *M* close to 1 in Eq. (2)) with a linear detection would exceed 200 GHz, well above the present state-of-the-art. Access to the source stochastic properties by evaluation of $g^{(2)}(\tau )$ from experimental temporal intensity traces^5^ fails in the present case.

### Non-linear detection

As explained earlier, stochastic field sources such as ASE are better described by the first and second order coherence functions $g^{(1)}(\tau )$ and $g^{(2)}(\tau )$. In practice, one could use the recorded temporal intensity waveform *I*(*t*) (see Fig. 5a) and compute $g^{(2)}(\tau )$ from its definition^5^ (see Eq. 6). Unfortunately, since the measurement of *I*(*t*) is distorted by the finite response of the detector, the computed $g^{(2)}(\tau )$ will obviously provide biased statistical properties. However, the direct measurement of $g^{(2)}(\tau )$ is indeed possible. In fact, short or ultrashort coherent pulses are commonly characterized by means of an intensity or an interferometric autocorrelator. The signal measured by an intensity autocorrelator is of the form:

10 $$\begin{aligned} S_{ac}(\tau ) \propto \int _{-\infty }^{\infty } |E(t)E^*(t-\tau )|^2 \, \mathrm {d}t \propto \int _{-\infty }^{\infty } I(t)I(t-\tau ) \, \mathrm {d}t \end{aligned}$$

It is straightforward to notice that $S_{ac}(\tau )$ is identically proportional to $g^{(2)}(\tau )$ defined in Eq. (6) offering a direct measurement of taa parhe statistical properties of the ASE source^27^. Two important points should be emphasized here. An intensity autocorrelator consists of a Michelson interferometer to set the delay between the 2 pulse replicas, a second-order non-linear stage and a receiver. As the sum frequency process induced in the non-linear crystal is instantaneous the generated beam carries the full statistical information of the source at times *t* and $t-\tau$ in an unbiased way. Then, the slow detector will simply integrate those fluctuations over time to give a mean value of the correlation at delay $\tau$. This is a very different situation if compared to the linear detection case seen above where the response of the “slow” detector was simply damping the fastest temporal structures of the field thus preventing the measurement of the full information. Measuring an autocorrelation trace of a CW stochastic beam might be cumbersome as the temporal structures peak power (reaching several times the average power as shown in Fig. 5b) would be of the order of a W. The pulsed ASE source presented here has been designed to provide a partially coherent beam with a fluctuating peak power potentially reaching tens of kW. We thus were able to utilize a commercial autocorrelator commonly used to characterize fs and ps pulses (see “Methods”).The following measurements have been performed at 13 W average power with a pulse repetition rate of 300 MHz. The optical gating was set to produce pulses of less than 50 ps. The pulsed ASE source coherence is adjusted by setting the spectral bandwidth in the seeder. Figure 6 displays recorded autocorrelation traces for spectral bandwidths ranging from 110 pm up to 660 pm. One can easily recognize the typical shapes (see Fig. 2) discussed in the theory part in “Methods” with a bell-type curve on which seats a coherence peak. We have then applied the parametrization presented earlier stating that the measured autocorrelation $S_{ac}(\tau )$ (identical to $g^{(2)}(\tau )$ when normalized to 2) can be decomposed in the product of $Q(\tau )$ with $(1+|g^{(1)}(\tau )|^2)$. As seen on Fig. 6 the parametrization is excellent if $Q(\tau )$ and $|g^{(1)}(\tau )|^2$ are assumed Gaussian. A slight distortion is however visible on the center plot where the autocorrelation is not fully symmetric (see Fig. 6b). This is due to the procedure of piecing up traces recorded with a 50 ps maximum delay autocorrelator to reach the full delay range of 150 ps potentially introducing inaccuracy. While $Q(\tau )$ remains rather constant, the coherence peak $|g^{(1)}(\tau )|^2$ gets narrower with wider spectra. The source characteristics have been extracted from the parametrization and are given in Table 1 for 4 different spectral bandwidth. $\Delta \tau _G$ corresponds to the FWHM of the pulse (carved temporal gate) and is deduced from the width of $Q(\tau )$ (autocorrelation width) assuming a Gaussian temporal gate. The coherence time $\tau _c$ of the pulsed stochastic source is extracted through 3 different methods. The first evaluation is a direct integration of $|g^{(1)}(\tau )|^2$ (Eq. 5). The second approach extract $\tau _c$ from a fit on Eq. (4). Finally, we have simulated the autocorrelation traces with the enhanced classical model. $\tau _c$ is given here as providing the best fit on the experimental data. First of all, the technique provides a robust evaluation of the pulse duration, which is identical in all cases, with a value around 41 ps. Note that the gate can be extended to longer pulses if necessary. Then, the 3 approaches to determine the coherence time give reasonably similar results for values ranging from 3.16 ps to 22.8 ps. A deviation is however noticeable for the largest bandwidth (shorter coherence time). This is due to the distortion of the amplified spectrum whose shape diverges from a Gaussian (one of the assumptions in the analytical development) and becomes triangular as can be seen on Fig. 6a.

**Table 1:** Retrieved pulsed ASE source parameters.

Measured bandwidth	Gate	Coherence peak integral		Coherence peak width		Classical model	
$\Delta \lambda$ [pm]	$\Delta \tau _G$ [ps]	$\tau _c$ [ps]	$\Delta \lambda$ [pm]	$\tau _c$ [ps]	$\Delta \lambda$ [pm]	$\tau _c$ [ps]	$\Delta \lambda$ [pm]
660	43.5	3.16	743	2.91	807	3.39	693
377	41.7	5.62	418	5.37	437	5.72	410
218	41.1	10.9	215	10.7	219	13.8	170
110	40.8	22.8	103	22.4	105	21.4	110

The pulse duration $\Delta \tau _G$ is deducted from the gate autocorrelation $Q(\tau )$ and the coherence time $\tau _{c}$ is evaluated through 3 approaches: integration of the coherence peak $\left( 1+|g^{(1)}(\tau )|^2\right)$ i.e. Eq. (5) (column 3), extraction from the coherence peak width (column 5) and best fit of the classical model (column 7). The corresponding ASE bandwidth is also given and can be compared to the measured spectral bandwidth.

### Enhanced SHG

**Figure 7:**
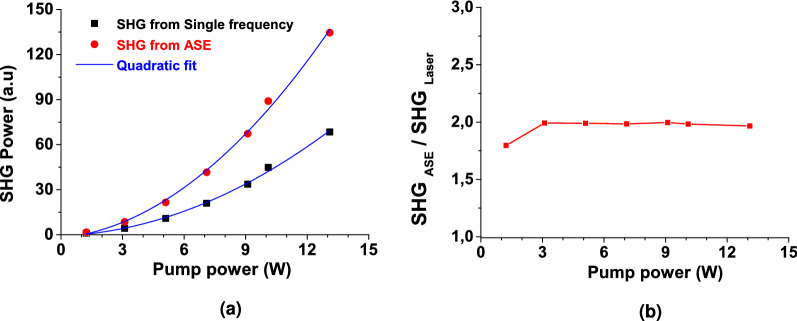
SHG of the pulsed partially coherent sources. (**a**) Black symbols: high coherence pulses. Red symbols: ASE pulses. Blue lines: quadratic fit. (**b**) SHG ratio between the high and low coherence pulses.

In this last part, we explore and demonstrate our system capabilities in terms of nonlinearity (“Enhancement of nonlinearities”). To this aim we compared second harmonic generation (SHG) in a nonlinear crystal successively pumped by high and low coherence pulses. The frequency conversion is achieved in a 5 mm BBO crystal (see Fig. 3). The high coherence source is obtained by injecting the single frequency laser in the Manny system (Fig. 3). Since the seed is continuous, the carved pulses of 41 ps are transform limited leading to a coherence time of 61.7 ps (a bandwidth of 38 pm). The partial coherence source is obtained by injecting the filtered ASE seeder. The case here corresponds to a coherence time of 5.6 ps (the 377 pm case in Table 1). All other parameters (average power, pulse duration, repetition rate and central wavelength) are kept identical. The converted power is compared on Fig. 7. As expected, the transform limited pulse case (high coherence) for which the peak intensity is well defined, follows a quadratic behaviour characterizing a second order non-linearity. A quadratic behaviour is also observed for the ASE source although the peak intensity is now a stochastic distribution. However, the power converted is significantly higher in the case of ASE as predicted by the theory (see Eq. 9). On Fig. 7b we have plotted the ratio of the measured SHG power for both cases. For all input average power, a factor of 2 is found in accordance with the theoretical value of 2!.

Finally in this proof of principle experiment, we have shown that ASE pulses are twice as efficient in wavelength conversion through SHG if compared to transform limited pulses with identical mean photon flux. This was known to be true for continuous wave sources but it still is valid for pulsed stochastic sources. While several tens or hundreds of W of instantaneous power is at reach in a continuous regime of ASE, powers of kW to GW can be envisioned in a pulsed regime depending on the pulse duration and the pulse repetition rate. It thus paves the way to the possibility of producing efficiently stochastic sources in the visible with adjustable coherence properties either in waveguides or in bulk materials.

## Conclusion

In this paper, we have presented a detailed study on the realisation of a high power partially coherent source based on an all-fibered pulsed ASE MOPA system. The system allows to tune the central wavelength (from 1020 to 1060 nm), the coherence time (from 3 to 23 ps), the average power (up to 13 W), the pulse length (from 40 ps to few ns), the repetition rate (from 5 MHz to 1 GHz) and lead to a complete control of the peak power random distribution. As the source characterization is no longer possible in the pulsed regime with a linear detection, we have proposed a diagnostic able to fully characterize the random properties of the pulsed ASE source by means of a non-linear detection method. Also, a classical model has been developed which provides the electric field matching the statistical properties of the experimental pulsed ASE source. Finally, we have demonstrated the expected factor of 2 enhancement of the SHG process when pumped by the pulsed ASE source. The latter openings the path to investigation of non-linear processes with such a source. For instance, according to theory (Eq. 9), four wave mixing (FWM, a third order non-linear process)^35^ using ASE pulses would require 6 times lower power to reach the generation threshold. Additionally, since the temporal coherence of the ASE pulses is controllable, one could use it to manage stimulated Raman scattering (SRS) or stimulated Brillouin scattering (SBS) in nanosecond pulses. More recently, we found an interest of high-energy low-temporal-coherence broadband light pulses in high-density physics, in particular for inertial confinement fusion (ICF)^36^.

## Methods

### Classical model for ASE

In this paper, we model the ASE much like what was previously documented^24^. We generate a vector S of $N = 2^{24}$ points representing the spectrum of the signal centered at 1030 nm ($f_{0} = 291.3$ THz). The frequency note increment $\delta f$ (10 kHz) is chosen so that the time window covers a duration of T = 100 ns. The actual spectrum is then populated by generating random numbers for the spectral phase (uniform distribution between 0 and 2$\pi$) and spectral amplitude (uniform distribution between 0 and 1) on a variable spectral window $\Delta f$, while the other components remain zero. $\Delta f$ can be varied between 0 and $\Delta f_{max}$ = min (N x 10 [kHz] / 2, $f_{0}$) to avoid spectral folding which could perturb the following statistical analysis as well as non-causal negative frequencies. The values used for this work (bandwidths less than 1 nm) are typically much smaller than $\Delta f_{max}$. The complex (non-dimensional) field *E*(*t*) is obtained by carrying out a Direct Fourier Transform (DFT) on S. The time increment is T/N = 0.95 fs. Afterwards, we calculate the time-domain intensity vector I as the square modulus of *E*(*t*).

The input signal of a photodiode is supposed proportional to the light intensity I, with a proportionality factor taken as 1 (non-essential for the following steps). To simulate an accurate voltage law $I_{filt}(t)$ at the output of the photodiode, I is filtered with the appropriate low-pass filter: I is first Fourier-transformed and its DFT is multiplied by the DFT of the impulse response of the photodiode and/or the oscilloscope. This impulse response is built as such: first, a linear ramp up to 1 during 150 fs (rise-time of the photodiode) then a slow exponential decrease towards 0 with a time constant corresponding to the cutoff frequency fc at -3 dB of the photodiode (typ. fc = 8 GHz in our experimental setup). The result is then inverse-Fourier-transformed to form the filtered response $I_{filt}(t)$. The numerical SPD of $I_{filt}(t)$, *P*(*I*), is presented in Fig. 1. It is obtained by computing the histogram of $I_{m} = I_{filt}(t) /\langle I_{filt} \rangle$ on 200 regularly-spaced bins, where $<>$ denotes here the numerical average, and normalizing the obtained vector by *NdIm* where *dIm* is the width of the bins. With this normalization, the numerical integration of $P(I)\cdot dI_{m}$ is equal to 1, as required for a SPD.

The second order coherence function (intensity autocorrelation) graphs presented in Fig. 2 are computed numerically as $F^{-1}(F(X)^2)$ where F is the DFT operator. For the continuous ASE, $X = I_{m}(t)$ and for the gate, $X = G(t)$.

As can be seen on Fig. 1, the model describes the Gaussian distribution of the ASE very accurately down to $P(I) \approx 10^{-6 }( I_{m} < 6)$. Below this value, the occurrences of very high overshoots become too seldom in the time sample (about 10 occurrences on a total of $N \approx 10^{6}$ points) to be accurately counted in the histogram.

As shown in Table 1, this model also accurately describes the coherence peak of the ASE. This suggests, although not entirely proves, that not only the distribution density but also the temporal behavior of *E*(*t*) is correctly simulated. Hence, in contrast with an approach exclusively based on the statistical description of *I*(*t*) starting from the degenerate expression of the Bose–Einstein distribution Eq. (1), this model describes the temporal phase of the noise, and can generate valid input conditions for the linear and nonlinear simulation of the propagation of the ASE in nonlinear simulation of the propagation of the ASE in fibers. The shape of the spectrum can be adapted by multiplying S with the desired profile, for instance a Gaussian curve of desired FWHM. As expected, the statistical behavior remains unchanged, and the coherence peak evolves correspondingly.

### Autocorrelation reconstruction method

The near-infrared (NIR) autocorrelator used is an “A.P.E Pulse Check” device with a measurable pulse width range from 50 fs to 35 ps. The pulse gating technology used in our set-up generates pulses of  40 ps. The pulses can therefore not be measured directly. Instead, the autocorrelation is measured piecewise setting the Michelson delay at 3 different positions around the zero delay and recording the signal over the scanning range paying attention to sufficient overlap. The full trace is then reconstructed by concatenation of the 3 partially overlapping scans. This manual process introduces inaccuracies leading to slightly asymmetric traces as in Fig. 6 (center) for instance (Supplementary information S1).

## Supplementary Information

Supplementary Information

## Data Availability

The data generated and/or analysed during the current study are available from the corresponding author upon request.

## References

[1] Orszag M (2000). Einstein’s theory of atom-radiation interaction, 1–9.

[2] Einstein A (1967). On the quantum theory of radiation. Old Quant. Theory.

[3] Loudon R (2000). The quantum theory of light.

[4] De Chatellus, H. G. & Pique, J. P. Coherence properties of modeless lasers. *Proc. Sci.* **101**. 10.22323/1.101.0008 (2009).

[5] Muniz-Cánovas P, Barmenkov YO, Kir’yanov AV, Cruz JL, Andrés MV (2019). ASE narrow-band noise pulsing in erbium-doped fiber amplifier and its effect on self-phase modulation. Opt. Exp..

[6] Goodman J (2000). Stat. Opt..

[7] Koichi S, Hidetosi T, Townes CH (1957). Fluctuations in amplification of quanta with application to maser amplifiers. J. Phys. Soc. Japan.

[8] Pietralunga SM, Martelli P, Martinelli M (2003). Photon statistics of amplified spontaneous emission in a dense wavelength-division multiplexing regime. Opt. Lett..

[9] Glauber RJ (1963). The quantum theory of optical coherence. Phys. Rev..

[10] Wong WS, Haus HA, Jiang LA, Hansen PB, Margalit M (1998). Photon statistics of amplified spontaneous emission noise in a 10-Gbit/s optically preamplified direct-detection receiver. Opt. Lett..

[11] Blazek M, Hartmann S, Molitor A, Elsaesser W (2011). Unifying intensity noise and second-order coherence properties of amplified spontaneous emission sources. Opt. Lett..

[12] Minguela-Gallardo JA, Barmenkov YO, Kir’yanov AV, Beltrán-Pérez G (2017). Photon statistics of actively Q-switched erbium-doped fiber laser. J. Opt. Soc. Am. B.

[13] Doronin IV (2019). Second-order coherence properties of amplified spontaneous emission. Opt. Exp..

[14] Muniz-Cánovas P, Barmenkov YO, Kir’yanov AV, Cruz JL, Andrés MV (2019). Ytterbium-doped fiber laser as pulsed source of narrowband amplified spontaneous emission. Sci. Rep..

[15] Xu J (2018). Exploration in performance scaling and new application avenues of superfluorescent fiber source. IEEE J. Select. Topics Quant. Electron..

[16] Clivaz X, Novàk RP, Gilgen HH, Marquis-Weible F, Salathé RP (1992). High-resolution reflectometry in biological tissues. Opt. Lett..

[17] Wysocki PF, Digonnet MJF, Kim BY, Shaw HJ (1994). Characteristics of erbium-doped superfluorescent fiber sources for interferometric sensor applications. J. Lightwave Technol..

[18] Shang Y (2016). Ultra-stable high-power mid-infrared optical parametric oscillator pumped by a super-fluorescent fiber source. Opt. Exp..

[19] Maestre H, Torregrosa AJ, Capmany J (2016). IR Image upconversion using band-limited ASE illumination fiber sources. Opt. Exp..

[20] Rao Y, Sarwade NP, Makkar R (2015). Modeling and simulation of Optical Coherence Tomography on Virtual OCT. Proc. Comput. Sci..

[21] Zhang, W., Fickler, R., Giese, E., Chen, L. & Boyd, R. W. Influence of pump coherence on the generation of position-momentum entanglement in optical parametric down-conversion. *Opt. Exp.* **27**, 20745. 10.1364/oe.27.020745 (2019). 1812.09532. 10.1364/OE.27.020745 31510163

[22] Arahira S, Murai H (2014). Wavelength conversion of incoherent light by sum-frequency generation. Opt. Exp..

[23] Jin A, Zhou H, Zhou X, Hou J, Jiang Z (2015). High-power ultraflat near-infrared supercontinuum generation pumped by a continuous amplified spontaneous emission source. IEEE Photon. J..

[24] Gorbunov OA, Sugavanam S, Churkin D (2014). Revealing statistical properties of quasi-CW fibre lasers in bandwidth-limited measurements. Opt. Exp..

[25] Kuusela TA (2017). Measurement of the second-order coherence of pseudothermal light. Am. J. Phys..

[26] Sala K, Kenney-Wallace G, Hall G (1980). CW autocorrelation measurements of picosecond laser pulses. IEEE J. Quant. Electron..

[27] Mussot A (2004). Spectral broadening of a partially coherent CW laser beam in single-mode optical fibers. Opt. Exp..

[28] Lecompte C, Mainfray G, Manus C, Sanchez F (1975). Laser temporal-coherence effects on multiphoton ionization processes. Phys. Rev. A.

[29] Spasibko KY (2017). Multiphoton effects enhanced due to ultrafast photon-number fluctuations. Phys. Rev. Lett..

[30] Dudley JM (2010). Extreme events in optics: Challenges of the MANUREVA project. Eur. Phys. J. Spec. Top..

[31] Chmela P (1985). Dependence of multi-photon absorption efficiency on photon statistics. Opt. Quant. Electron..

[32] Prantil MA (2013). Widely tunable 11 GHz femtosecond fiber laser based on a nonmode-locked source. Opt. Lett..

[33] Aubourg A, Lhermite J, Hocquet S, Cormier E, Santarelli G (2015). Generation of picosecond laser pulses at 1030 nm with gigahertz range continuously tunable repetition rate. Opt. Lett..

[34] Metcalf, A. J., Fredrick, C. D., Terrien, R. C., Papp, S. B. & Diddams, S. A. 30 GHz electro-optic frequency comb spanning 300 THz in the near infrared and visible. *Opt. Lett.* **44**, 2673. 10.1364/ol.44.002673 (2019). 1902.02817.

[35] Delagnes J-C (2018). High-power widely tunable ps source in the visible light based on four wave mixing in optimized photonic crystal fibers. Opt. Express.

[36] Cui Y (2019). High-energy low-temporal-coherence instantaneous broadband pulse system. Opt. Lett..

