# Supplementary material for: High-power amplified spontaneous emission pulses with tunable coherence for efficient non-linear processes
Source: Sci Rep. 2021 Mar 1;11:4844. doi: 10.1038/s41598-021-83443-2 (PMC7921131; doi:10.1038/s41598-021-83443-2)
Supplement: Supplementary file 1 — Supplementary Information [file 41598_2021_83443_MOESM1_ESM.pdf]

SCIENTIFIC REPORTS: Supplementary Document

Article Title: High-power amplified spontaneous emission pulses with tunable coherence for efficient non-linear processes

Authors: *Nicolas Valero*[1+,*], *Denis Marion*[1,+], *Jerome Lhermite*[1,+], *Jean – Christophe Delagnes*[1], *William Renard*[4], *Romain Royon*[4], *Eric Cormier*[1,2,3].

Affiliations:

[1] - Centre des Lasers Intenses et Applications (CELIA), UMR 5107, Université de Bordeaux, CNRS, CEA – Talence, France.

[2] - Institut Universitaire de France (IUF), 1 rue Descartes,75231 Paris Cedex 05.

[3] - Laboratoire Photonique, Numerique et Nanoscience (LP2N), Institut d'Optique Graduate School-CNRS-Universite Bordeaux, rue F. Mitterrand, F–33400, Talence, France.

[4] - IRISIOME, rue F. Mitterrand, F–33400, Talence, France .

[*] - Corresponding author: nicolas.valero@u-bordeaux.fr.

[+] - Equal contribution : Nicolas Valero, Denis Marion, Jerôme Lhermite.

Second order coherence function in the case of a gated stochastic source

Derivation of $g^{(2)}$ in the case of a gated stochastic source

Considering two statistically independent random variables X and Y, we can write

$$E(X \cdot Y) = E(X)E(Y) \tag{1}$$

where $E(X)$ designates the expected value of variable X. The intensity of a gated ASE source can be written as $I(t) = G(t) \cdot I_{\mathrm{ASE}}(t)$. $G(t)$ is comprised between 0 and 1 and stands for the deterministic gate-function (e.g., the transmission function of a modulator driven by a pulse generator). $I_{\mathrm{ASE}}(t)$ represents the statistically ergodic, random intensity delivered by a "continuous" ASE source.

The second-order coherence function $g^{(2)}_{\mathrm{gated}}(\tau)$ of the random variable $I(t)$ is defined mathematically as:

$$\begin{aligned} g^{(2)}_{\mathrm{gated}}(\tau) &= \lim_{T\to+\infty}\left(\frac{1}{T}\right)\int_{-T}^{+T} G(t)I_{\mathrm{ASE}}(t)G(t+\tau)I_{\mathrm{ASE}}(t+\tau)dt \\ &= \lim_{T\to+\infty}\left(\frac{1}{T}\right)\int_{-T}^{+T} G(t)G(t+\tau)I_{\mathrm{ASE}}(t)I_{\mathrm{ASE}}(t+\tau)dt \\ &= E(X_\tau \cdot Y_\tau) \end{aligned} \tag{2}$$

where the random variables $X_\tau(t) = G(t)G(t+\tau)$ and $Y_\tau(t) = I(t)I(t+\tau)$ are statistically independent (even though X_τ is actually deterministic). We may then apply the relation given in Eq. 1 and write:

$$g^{(2)}_{\mathrm{gated}}(\tau) = \lim_{T\to+\infty}\left(\frac{1}{T}\right)\int_{-T}^{+T} G(t)G(t+\tau)dt \cdot \lim_{T\to+\infty}\left(\frac{1}{T}\right)\int_{-T}^{+T} I_{\mathrm{ASE}}(t)I_{\mathrm{ASE}}(t+\tau)dt$$

or

$$g^{(2)}_{\mathrm{gated}}(\tau) = Q(\tau) \cdot g^{(2)}_{\mathrm{ASE}}(\tau) \tag{3}$$

where $Q(\tau)$ is the normalized intensity correlation function of the time gate-function G and $g^{(2)}_{\mathrm{ASE}}$ is the second-order coherence function of the continuous, ergodic ASE, although Eq. 3 is actually general and stands for all sorts of gated stochastic light emissions. From a physical point of view, Eq. 3 reveals the relation between the auto-correlation traces of the gated and the continuous ASE sources. In our case, as ASE is a Gaussian random phenomenon, we may derive even further [1] and express $g^{(2)}_{\mathrm{gated}}$ in relation with $g^{(1)}_{\mathrm{ASE}}$:

$$g^{(2)}_{\mathrm{gated}}(\tau) = Q(\tau) \cdot \left(1 + |g^{(1)}_{\mathrm{ASE}}(\tau)|^2\right) \quad (4)$$

References

[1] H. Guillet de Chatellus, J. P. Pique. *Coherence properties of modeless lasers.* PoS – Proceedings of Science, SISSA, 2009, 09 (08). hal-00952625
